# Dental Pain in Children with Intellectual Disabilities: Caregivers' Perspective

**DOI:** 10.1155/2012/701608

**Published:** 2012-08-14

**Authors:** Sumer M. Alaki, Niveen S. Bakry

**Affiliations:** Faculty of Dentistry, King Abdulaziz University, P.O. Box 80209, Jeddah 21589, Saudi Arabia

## Abstract

*Purpose*. Description of pain is generally difficult in children, and more so in those with intellectual disabilities (ID). *Aim*. This study is aimed at evaluating dental pain from caregivers' perspective in children with ID. *Methods*. The study sampled 86 children (33 with ID, 53 normally developing) ages ranges from birth to 16 years old among those visiting the School of Dentistry, King Abdulaziz University, Saudi Arabia. Caregivers were asked about their awareness of dental pain in their wards using the Dental Discomfort Questionnaire (DDQ+). The children were examined for dental caries and periodontal status. *Results*. The mean DDQ+ in children with ID (4.55 ± 3.46) was not significantly different from that in healthy children (4.19 ± 3.26, *P* = 0.7). Children with ID had more salivation (*P* = 0.01) and were putting their hands inside their mouths more often (*P* = 0.003). *Conclusions*. Caregivers can recognize dental pain-related behaviors in children with ID such as excessive salivation and putting hands inside the mouth more often.

## 1. Introduction

Since pain is an inherently subjective phenomenon, it is often said that the “gold standard” for pain assessment in both children, and adults is verbal-reporting [1–3]. That is to say that patient's verbal reporting of pain is the only way to determine its presence, intensity, and quality [4]. However, reliable description of pain may be difficult for certain individuals including infants, very young children, individuals with intellectual disabilities (ID) and adults with severe dementia. 

Reliable description of pain may be difficult for children due to their cognitive immaturity and their inability to separate pain from fear and anxiety [5]. Children with ID frequently have the added challenge of not being able to express their pain or verbalize it. This explains why these children were believed not to experience pain and were forced to undergo painful procedures on occasions without the proper control of pain [6]. It also explains why improper pain assessment and management have been practiced with individuals with ID [7]. 

Intellectual disability which affects nearly 2.5% of the population is currently defined by the American Association on Intellectual and Developmental Delay (AAIDD) as significantly subaverage intellectual functioning, existing concurrently with related limitations in two or more of certain adaptive skills including communication, self-care and social skills manifesting before the age of 18 years [8, 9]. Research shows that children with developmental disabilities generally experience more pain than normal children [10, 11] due to comorbid conditions associated with their disability or due to medical interventions necessary to monitor or treat the disability [12]. Their pain however may not be properly appreciated because of their lack of verbal communication and because many of these children often have idiosyncratic behaviors such as moaning, grunting, and grimacing which may lead to overestimation of pain by those unfamiliar with the child [13]. Breau et al. (2003) showed that children who experienced the most pain were those who were least able to verbally describe it, that is, children with greater physical and cognitive disability [14]. 

 Studies show that children with disabilities have higher dental caries experience, unmet treatment needs, and prevalence of malocclusion, than those without disabilities [15–17]. In their study of special needs children, Hennequin et al. (2000) showed that there was a significant underestimation of dental treatment needs by parents and dentists suggesting that dental pain in this population was also underestimated [18].

 This study was aimed at assessing the ability of caregivers of children with ID to evaluate the presence of dental pain through behavioral changes in their children.

## 2. Participants and Methods

### 2.1. Participants

This study recruited a total of 86 children among those visiting the dental clinics at the Faculty of Dentistry, and the hospital dental clinics at King Abdulaziz University (KAU), Jeddah, Saudi Arabia, during the years 2009 and 2010. Recruited children were divided into cases and controls. Cases fulfilled the following criteria.

1. Both boys and girls ages range from birth to 16 years.
2. A history of ID as verified by the child's medical file or caregivers' reporting.
3. Child may be verbal or nonverbal.
4. Caregivers were able to understand spoken Arabic or English.
5. Included children could be diagnosed with medical problems other than ID.

Controls included age-matched children recruited from the dental clinics at the Faculty of Dentistry, and the hospital dental clinics at King Abdulaziz University (KAU). They basically fulfilled the same criteria only they had normal intellectual development without ID.

### 2.2. Methods

Before the beginning of the study, ethical approval was obtained from the Faculty of Dentistry, KAU, to conduct the study. The investigators developed a questionnaire containing a mixture of closed- and open-ended questions regarding participants' demographical, medical, and dental data. Two investigators were calibrated to do intra- and extraoral examinations on children. Reliability of the two examiners was assessed in measuring dental caries on 10% of the sampled children with Kappa = 0.85, *P* = 0.0001. The investigators reviewed the children's medical records to determine their medical diagnosis, prescribed medications and degree of ID (if present in medical record). The nature of the study was explained to the caregivers and signed written consents were obtained. The investigators pretested the questionnaire prior to the study to check its readability and caregivers' understanding of questions. The investigators then interviewed caregivers and assisted them in filling out the questionnaire. 

Dental pain was measured using the “Dental Discomfort Questionnaire” (DDQ+) [19]. This questionnaire was originally developed by Versloot et al. [19] to measure possible dental pain-related behaviors in very young children and was subsequently modified to include children with learning disabilities. Behaviors such as crying with meals or at night, problems of chewing or brushing, excessive salivation, or putting hands inside the mouth were among those included in the DDQ+ (Table 2). Studies show that the behaviors included in the questionnaire were observed more often in children suffering from caries and toothache than in children with only caries or in children without caries or toothache. The questionnaire was filled out by the investigators during their interviews with caregivers. The investigators used Arabic or English languages to describe the items of the questionnaire according to the native language of the caregiver.

For each question in the DDQ+, parents' rated the occurrence of the behavior as “never” coded as zero, “sometimes” coded as 1, or “frequently” coded as 2. A total numeric DDQ+ for each child and a mean DDQ+ for the group were calculated. Caregivers and investigators were also asked to rate the children's dental pain on a ten-point scale where zero represented the absence of pain and ten the maximum pain imaginable [20]. This scale was converted to an ordinal scale with mild, moderate, and severe categories. 

Dental examinations were done on the dental chair with optimal lighting and intraoral mirror. Gentle removal of soft debris on teeth was done when needed without forceful use of dental explorer. Caries examination was based on the “WHO” criteria where the DFT/dft scores were measured [21]. Caries was also evaluated on a yes-no basis. Dental caries was considered at the level of frank cavitation which was defined as a break of 0.5 mm or more into enamel. Incipient or questionable caries were not included in the DFT/dft scores. Filled teeth were defined as those with any type of restorations excluding sealants. Because many included children were at the age of mixed dentition, the missing “m” component of the dmft was not included. 

The severity of dental treatment needs was determined for each child based on the following categories. None: no restorative treatment required; simple: preventative treatment required such as scaling, prophylaxis, oral hygiene instructions, topical fluoride application, or sealants; moderate: one or more teeth requiring one- or two-surface restorations; complex: one or more teeth requiring a three- or four-surface restorations, stainless steel crowns, endodontic therapy, or extractions [15].

### 2.3. Statistical Analysis

The statistical analysis was done using Windows SPSS software version 15. Bivariate analyses were done to study associations between demographic, medical, and dental data and the DDQ. Regression models were also used with the main outcome variable being the average total DDQ score. The level of significance (*α*) was set at 0.05.

## 3. Results

### 3.1. Children's Demographics

The study sampled a total of 86 children of which 33 were cases diagnosed with ID and 53 were healthy age-matched controls. The sample contained 44 (51.16%) males and 42 (48.84%) females. The sampled children were categorized into three age groups; birth to 6 years (*n* = 29), 6 to 12 years (*n* = 53), and 12 to 16 years (*n* = 4). No significant difference was found between cases and controls with regards to demographical data including age (*x* ^2^ = 3.92, *P* = 0.4), gender (*x* ^2^ = 0.25, *P* = 0.62), and mother's education (*x* ^2^ = 2.05, *P* = 0.56) or occupation (*x* ^2^ = 0.37, *P* = 0.54). A significant association however was found between father's education and having a child with ID (*x* ^2^ = 8.46, *P* = 0.04). Mothers were the caregivers answering the questionnaires in the majority of participating children (73.6% in healthy children and 84.4% in children with ID).

### 3.2. Medical Data

Among children with ID, 31% were perceived by their parents to have mild delays, 37.9% moderate, and 24.1% severe delays, while 6.8% of parents did not recognize the presence of delay despite the presence of a confirmed diagnosis in the child's medical record. The majority of children with ID were diagnosed at birth (84.8%). The results showed that 97% of children with ID had other associated medical problems compared to 26.4% of healthy children (*P* = 0.0001), and that 57.6% were taking medications compared to 13.2% of healthy children (*x* ^2^ = 18.89, *P* = 0.0001). 

 When asked about the child's verbal abilities, 57.6% of parents with children having ID replied that their children could speak. Among those, 74% (*n* = 14) were perceived to have partly intelligible speech and 26% (*n* = 5) very intelligible speech. Children with ID had significantly higher prevalence of heart (*P* = 0.05) and respiratory (*P* = 0.05) conditions, growth problems (*P* = 0.03), seizures (*P* = 0.0001), and verbal and physical limitations (*P* = 0.0001 and 0.007, resp.).

### 3.3. Dental Findings

Table 1 shows a comparison of the DFT and dft scores in healthy controls and those with ID. It can be seen that the DFT score was significantly higher in children with ID (*P* = 0.04) and that these children had higher D component compared to that in healthy children (*P* = 0.03). Results show no difference in the F component between the two groups. The dft score on the other hand, was higher in healthy children (*P* = 0.04) and so was its d component (*P* = 0.05).

### 3.4. Pain Data

Table 2 shows caregivers' and investigators' reporting of children's dental pain. The results essentially show that caregivers' perception of dental pain was comparable in healthy children and those with ID. The investigators also showed no significant difference in their perception of dental pain among the two groups. The investigators however, perceived more children to have dental pain compared to caregivers and rated the pain higher on the pain scale. 

When caregivers were asked about specific behaviors related to dental pain (DDQ+) results showed that the mean DDQ+ in healthy children was 4.19 ± 3.26 and in children with ID was 4.3 ± 3.5 with no significant difference (Mann-Whitney = 0.08, *P* value = 0.94). Also, the mean DDQ+ in children with ID who had dental caries (4.55 ± 3.46) was not significantly different from that in healthy children (4.19 ± 3.26) with dental caries (*P* = 0.7).  Table 3 shows elements of the DDQ+ in children with dental caries (healthy versus those with ID). The table shows that children with ID had significantly more salivation (*P* = 0.01) and were putting their hands inside their mouths more often (*P* = 0.003). Healthy children with dental caries; however, tended to chew more on one side of the mouth (*P* = 0.04). The assessment of dental pain by caregivers and investigators was compared in healthy children and those with ID to check for agreement (Table 4). The results showed significant agreement between caregivers and investigators in both groups of children being more statistically significant in healthy children (*P* = 0.0001). 

 The analysis looked at the correlation between the average DDQ+ and the DFT/dft scores. The results showed a significant positive correlation in healthy children between the DDQ+ and DFT score (Spearman rho = 0.47, *P* = 0.02), but no correlation was found between the DDQ+ and dft (*P* = 0.11). On the other hand, in children with ID, a significant positive correlation was found between the DDQ+ and dft (Spearman rho = 0.47, *P* = 0.01), but no correlation was found between the DDQ+ and the DFT (*P* = 0.3). 

 The association between caregivers' perception of the presence of dental pain and the complexity of dental treatment needs was evaluated (Table 4). The results showed a stronger association between the ability of caregivers to determine pain and the complexity of treatment in children with ID than healthy children (*P* = 0.003) as seen in Table 5. Additionally, caregivers of children with ID were more able to determine severity of pain (mild, moderate, severe) with relation to complexity of treatment than caregivers of healthy children (*P* = 0.003).

The regression model with the DDQ+ as the outcome variable did not show any of the predictors (gender, mother's education or occupation, father's education, child's medical condition, and medication intake) to be significant.

## 4. Discussion

Reliable description of pain is generally difficult in children, and more so in those with ID due to their cognitive immaturity, lack of verbal skills, and their demonstration of idiosyncratic behaviors [5, 13]. Research shows that children with disabilities are generally at higher risk of having their pain underestimated or undertreated [22].

Due to communication difficulties, children with ID often depend on their parents and caregivers in discerning the presence of pain. A study by Hennequin et al. in 2003 showed that parents had more difficulties recognizing pain in children with Down syndrome than healthy children [23]. Also, children with ID often attended dental visits when symptoms of acute dental pain arise [24, 25]. Research shows a relationship between certain pain-associated behaviors and the presence of toothache and dental caries with children having dental caries or tooth ache displaying these behaviors more often [19].

 Our results show that the DFT score was significantly higher in children with ID whereas the dft was higher in healthy children. This was surprising as children with ID seemed to have more caries risk factors such as associated medical conditions and intake of medications. Our assumption is that children with ID had both higher DFT and dft scores compared to healthy children. However, because of their often uneasy behavior in dental settings, it is can be difficult to provide them with good quality dentistry which may at times make extractions a common treatment. Because our calculations of the DFT/dft scores did not include the missing teeth component, we may have underestimated the actual caries severity (dft score) in children with ID. This underestimation was not seen in the DFT score because children in our sample were mostly 6–12 years old and may not have had extractions of permanent teeth yet. 

Our data indicate that caregivers can recognize pain-related behaviors in children with ID such as excessive salivation and putting hands inside the mouth more often. This finding is in accord with previous research and indicates that parents become experts in their children's behavior; hence there are useful tools to healthcare providers in making diagnoses [19]. The positive correlation between average DDQ and DFT/dft shows that the greater the severity of dental caries, the more likely the child will display pain-related behaviors, again a finding in accord with previous literature [26]. These findings should alert both caregivers' and dentists that determining the presence of dental pain in children with ID is a joint process between the two.

This study had some limitations which can be addressed in future studies. Some of these limitations included the lack of a professional assessment of the level of intellectual disability. The investigators evaluated the medical histories of all children from their medical records. However, a professional psychological assessment of their level of disability was not always present. Future research in this area can be modified to include only children with professional psychological assessment and to stratify the analysis by level of intellectual disability.

## 5. Conclusions

Based on the investigators findings from this research, the following can be concluded.

1. There is no significant difference in the general display of dental pain-related behaviors among healthy children and those with ID.
2. Children with ID display more salivation and putting hands inside the mouth in response to dental pain.
3. The more severe the carious process the more the child will display pain-related behaviors.
4. Caregivers of children with ID who had complex dental treatment needs were more able to detect dental pain in their children compared to those of healthy children.

## Figures and Tables

**Table 1:** DFT and dft scores in healthy children and children with ID.

DFT/dft scores	Healthy children	Children with ID	Mann-Whitney test	*P* value
DFT	0.92	2.32	2.09	0.04^∗^
D	0.77	2.04	2.18	0.03^∗^
F	0.15	0.28	0.53	0.6

dft	8.83	6.81	2.07	0.04^∗^
d	8.32	6.47	1.94	0.05^∗^
f	0.36	0.34	0.03	0.97

^∗^Statistically significant at *α* = 0.05.

**Table 2:** Caregivers' and investigators' reporting of children's dental pain.

Questions to caregivers				
Variables		Healthy children *n* (%)	Children with ID *n* (%)	*x* ^2^ *P* value
Do you think your child has dental pain?	No	12 (22.6)	6 (20.7)	0.04
	Yes	41 (77.4)	23 (79.3)	0.84

How frequent is your child's dental pain?	Never	12 (22.6)	7 (24.1)	0.3
	Occasionally	27 (50.9)	13 (44.8)	0.86
	Always	14 (26.4)	9 (31)	

How severe is your child's dental pain?^∗^	Mean ± SD	4.1 ± 3.07	4.36 ± 3.05	0.35
	Median	4	5	0.73

When does pain mostly occur?	During meals	29 (70.7)	14 (66.7)	
	During daytime	1 (2.4)	0	0.76
	At night	5 (12.2)	3 (14.3)	0.86
	Multiple occasions	6 (14.6)	4 (19)	

Questions to investigators				
Variables		Healthy children *n* (%)	Children with ID *n* (%)	*x* ^2^ *P* value

Do you think this child has dental pain? ^†^	No	5 (9.4)	1 (3.1)	0.4
	Yes	48 (90.6)	31 (96.9)	

How severe is this child's dental pain? ^††^	Mean ± SD	6.3 ± 2.87	6.78 ± 2.7	0.78
	Median	7	7	0.43

^∗^Mann-Whitney *U* test used for comparison.

^†^Fisher exact test used for comparison.

^††^ *t*-test used for comparison.

**Table 3:** Comparison of DDQ items between healthy children and children with ID in groups with dental caries.

Items of the DDQ	Frequency	Healthy children *n* (%)	Children with ID *n* (%)	*x* ^2^	*P* value
Problems with brushing upper teeth	Never	44 (83)	24 (80)	0.35	0.84
	Occasionally	2 (3.8)	2 (6.7)		
	Always	7 (13.2)	4 (13.3)		

Problems with brushing lower teeth	Never	44 (83)	25 (83.3)	0.28	0.87
	Occasionally	3 (5.7)	1 (3.3)		
	Always	6 (11.3)	4 (13.3)		

Puts away something nice to eat	Never	42 (79.2)	20 (64.5)	2.42	0.3
	Occasionally	3 (5.7)	4 (12.9)		
	Always	8 (15.1)	7 (22.6)		

Bites with molar instead of front teeth	Never	47 (88.7)	26 (89.7)	0.57	0.75
	Occasionally	1 (1.9)	0 (0)		
	Always	5 (9.4)	3 (10.3)		

Chewing at one side	Never	34 (65.4)	26 (89.7)	6.26	0.04^∗^
	Occasionally	2 (3.8)	1 (3.4)		
	Always	16 (30.8)	2 (6.9)		

Problems chewing	Never	37 (71.2)	21 (72.4)	0.02	0.99
	Occasionally	2 (3.8)	1 (3.4)		
	Always	13 (25)	7 (24.1)		

Reaching for the cheek while eating	Never	28 (52.8)	18 (62.1)	0.67	0.71
	Occasionally	4 (7.5)	2 (6.9)		
	Always	21 (39.6)	9 (31)		

Crying at night	Never	42 (79.2)	22 (71)	1.6	0.45
	Occasionally	3 (5.7)	1 (3.2)		
	Always	8 (15.1)	8 (25.8)		

Crying during meals	Never	40 (75.5)	23 (76.7)	5.73	0.06
	Occasionally	7 (13.2)	0 (0)		
	Always	6 (11.3)	7 (23.3)		

Earache at night	Never	51 (96.2)	33 (100)	1.2	0.55
	Occasionally	1 (1.9)	0 (0)		
	Always	1 (1.9)	0 (0)		

Earache at daytime	Never	52 (98.1)	33 (100)	—	1.00
	Occasionally	1 (1.9)	0 (0)		
	Always	0 (0)	0 (0)		

Earache during eating	Never	53 (100)	33 (100)	—	—
	Occasionally	0 (0)	0 (0)		
	Always	0 (0)	0 (0)		

Excessive salivation	Never	52 (98.1)	25 (80.6)	—	0.01^∗^
	Occasionally	0 (0)	0 (0)		
	Always	1 (1.9)	6 (19.4)		

Putting hand inside mouth	Never	47 (88.7)	21 (70)	11.45	0.003^∗^
	Occasionally	6 (11.3)	3 (10)		
	Always	0 (0)	6 (20)		

^∗^Statistically significant at *α* = 0.05.

**Table 4:** Agreement between pain assessments (yes/no) by caregivers and investigators.

Dentist reporting of presence of pain	Parent-reporting of presence of pain			
	Healthy children		Children with ID	
	No	Yes	No	Yes
	*n* (%)	*n* (%)	*n* (%)	*n* (%)
No	5 (41.7)	0	1 (20)	0
Yes	7 (58.3)	41 (100)	4 (80)	23 (100)
Kappa	0.53		0.29	
*P* value	0.0001^∗^		0.03^∗^	

^∗^Statistically significant at *α* = 0.05.

**Table 5:** Association between dental treatment needs and caregivers' ability to recognize presence of pain.

		Does child have pain (question to caregiver)?			
Type of subject	Child's dental treatment needs	No	Yes	Total	Kendall's tau-b *P* value
		*n* (%)	*n* (%)		
Healthy children	Non/simple	0 (0)	0 (0)	0 (0)	
	Moderate	4 (33.3)	2 (4.9)	6 (11.3)	0.38
	Complex	8 (66.7)	39 (95.1)	47 (88.7)	0.06
	Total	12 (100)	41 (100)	53 (100)	

Children with ID	Non/simple	3 (50)	0 (0)	3 (10.3)	
	Moderate	2 (33.3)	1 (4.3)	3 (10.3)	0.79
	Complex	1 (16.7)	22 (95.7)	23 (79.3)	0.003^∗^
	Total	6 (100)	23 (100)	29 (100)	

^∗^Statistically significant at *α* = 0.05.

## References

[1] Merskey H (1986). Classification of chronic pain: description of chronic pain syndromes and definitions of pain terms. *Pain Supplement*.

[2] Merskey H, Bogduk N (1994). *Classification of Chronic Pain: Description of Chronic Pain Syndromes and Definitions of Pain Terms*.

[3] McIntosh N (1997). Pain in the newborn, a possible new starting point. *European Journal of Pediatrics*.

[4] Meinhart NT, McCaffery M (1983). *Pain: A Nursing Approach to Assessment and Analysis*.

[5] Franck LS, Greenberg CS, Stevens B (2000). Pain assessment in infants and children. *Pediatric Clinics of North America*.

[6] Galton F (1907). *Inquiries into Human Faculty and Its Development*.

[7] Malviya S, Voepel-Lewis T, Tait AR (2001). Pain management in children with and without cognitive impairment following spine fusion surgery. *Paediatric Anaesthesia*.

[8] American psychiatric Association. http://www.psych.org/.

[9] Luckasson R, Coulter DL, Polloway EA (1992). *Mental Retardation: Definition, Classification, and Systems of Supports*.

[10] Breau LM, Camfield CS, McGrath PJ, Finley GA (2003). The incidence of pain in Children with severe cognitive impairments. *Archives of Pediatrics and Adolescent Medicine*.

[11] Stallard P, Williams L, Lenton S, Velleman R (2001). Pain in cognitively impaired, non-communicating children. *Archives of Disease in Childhood*.

[12] Hadden KL, Von Baeyer CL (2002). Pain in children with cerebral palsy: common triggers and expressive behaviors. *Pain*.

[13] Fanurik D, Koh JL, Schmitz ML, Harrison RD, Conrad TM (1999). Children with cognitive impairment: parent report of pain and coping. *Journal of Developmental and Behavioral Pediatrics*.

[14] Breau LM, MacLaren J, McGrath PJ, Camfield CS, Finley GA (2003). Caregivers’ beliefs regarding pain in children with cognitive impairment: relation between pain sensation and reaction increases with severity of impairment. *Clinical Journal of Pain*.

[15] Desai M, Messer LB, Calache H (2001). A study of the dental treatment needs of children with disabilities in Melbourne, Australia. *Australian Dental Journal*.

[16] Pope JE, Curzon ME (1991). The dental status of cerebral palsied children. *Pediatric Dentistry*.

[17] Ackerman A, Wiltshire WA (1994). The occlusal status of disabled children. *The Journal of the Dental Association of South Africa*.

[18] Hennequin M, Faulks D, Roux D (2000). Accuracy of estimation of dental treatment need in special care patients. *Journal of Dentistry*.

[19] Versloot J, Veerkamp JSJ, Hoogstraten J (2006). Dental Discomfort Questionnaire: Assessment of dental discomfort and/or pain in very young children. *Community Dentistry and Oral Epidemiology*.

[20] Scott J, Huskisson EC (1976). Graphic representation of pain. *Pain*.

[21] World Health Organization (1997). *Oral Health Survey: Basic Methods*.

[22] McGrath PJ, Finley GA, McGrath PJ (1998). Behavioral measures of pain. *Measurement of Pain in Infants and Children*.

[23] Hennequin M, Faulks D, Allison PJ (2003). Parents’ ability to perceive pain experienced by their child with down syndrome. *Journal of Orofacial Pain*.

[24] Owens PL, Kerker BD, Zigler E, Horwitz SM (2006). Vision and oral health needs of individuals with intellectual disability. *Mental Retardation and Developmental Disabilities Research Reviews*.

[25] Gizani S, Declerck D, Vinckier F, Martens L, Marks L, Goffin G (1997). Oral health condition of 12-year-old handicapped children in Flanders (Belgium). *Community Dentistry and Oral Epidemiology*.

[26] Versloot J, Versloot J (2007). The Dental Discomfort Questionnaire: its use with mentally disabled children. *Pain in Pediatric Dentistry*.

